# Effects of different doses of oregano essential oil on growth performance, health parameters, and the gut microbiome-metabolome profile in heat-stressed Pinan cattle (Piedmontese × Nanyang)

**DOI:** 10.3389/fmicb.2025.1712904

**Published:** 2026-01-29

**Authors:** Jiangge Wang, Lei Liu, Jiashun Sheng, Liyang Zhang, Qiaozhen Li, Tengyun Gao, Xian Liu

**Affiliations:** 1 Henan International Joint Laboratory of Nutrition Regulation and Ecological Raising of Domestic Animal, College of Animal Science and Technology, Henan Agricultural University, Zhengzhou, China; 2 Animal Husbandry Bureau of Xinye County, Xinye, China; 3 Henan Animal Husbandry Technology Extension Station, Zhengzhou, China

**Keywords:** heat stress, metabonomics, microbiota, oregano essential oil, Pinan cattle (Piedmontese × Nanyang)

## Abstract

**Introduction:**

This study investigated the dose–response efficacy of dietary oregano essential oil (OEO) in mitigating severe heat stress (THI ≈ 86) in beef cattle.

**Methods:**

Thirty-six Pinan bulls were fed a basal diet alone (control) or supplemented with 7 (L-OEO) or 14 g/d (H-OEO) of OEO for 60 days.

**Results:**

The low-dose OEO (7 g/d) significantly improved hepatic function (reduced ALT, AST; increased ALB, TP), enhanced immune (increased IgA, IgM) and antioxidant status (decreased MDA, increased SOD, CAT), and increased the ruminal abundance of *Bacteroidota* and *Prevotella*, which correlated negatively with acyl-glycine metabolites. In contrast, the high dose (14 g/d) only increased GSH-Px and T4, resulted in higher MDA than L-OEO, and did not significantly affect the rumen microbiota.

**Conclusion:**

Supplementation with 7 g/d OEO optimally improved overall health and metabolic function in heat-stressed bulls, whereas a 14 g/d dose offered no additional benefits.

## 1 Introduction

Summer heat stress poses a significant challenge to global beef production. Beef cattle are particularly susceptible due to the metabolic heat generated from ruminal fermentation and their insulating hair coats (Nakamura and Morrison, 2010). When the temperature-humidity index (THI) exceeds a critical threshold, cattle exhibit characteristic signs of heat stress, including reduced feed intake and impaired ruminal function (Mader et al., 2006). To maintain thermal homeostasis, physiological responses are activated, such as the suppression of the hypothalamic feeding center to reduce appetite and minimize endogenous heat production from feeding and rumination (Morrison et al., 2014; Bernabucci et al., 2010).

This systemic stressor profoundly disrupts the gastrointestinal ecosystem. Heat stress induces ruminal and intestinal dysbiosis through a combination of elevated core body temperature, reduced feed intake, and altered water consumption (Bernabucci et al., 2010; Zhao et al., 2019; Park et al., 2022). This shift often depletes beneficial cellulolytic bacteria while enriching potential pathogens and lactate producers, compromising volatile fatty acid profiles, fiber digestibility, and gut barrier integrity (Bernabucci et al., 2010; He et al., 2021). The resulting endotoxin translocation and inflammatory response exacerbate systemic metabolic dysregulation.

As the central metabolic organ, the liver is severely impaired under heat stress. It faces the dual challenge of maintaining gluconeogenesis while counteracting oxidative damage (Bernabucci et al., 2010; Abbas et al., 2020). Persistent oxidative stress inhibits fatty acid oxidation and export, leading to hepatic lipidosis (Skibiel et al., 2018). Concurrently, mitochondrial dysfunction increases reactive oxygen species (ROS) production, while key antioxidant enzymes (e.g., SOD, GSH-Px, CAT) are depleted, culminating in hepatocellular injury (Wang et al., 2020; Eslamizad et al., 2024).

Given this multifaceted pathophysiology, natural interventions with multi-target effects are needed. Oregano essential oil (OEO), rich in bioactive phenolics like thymol and carvacrol, shows promise in mitigating heat stress in livestock (Garcia-Galicia et al., 2020; Nazar et al., 2019; Yousefi et al., 2024). Its potential mechanisms include modulating gut microbiota, alleviating oxidative stress, and regulating immune responses (Cui et al., 2024; Habibi et al., 2015; Terenina et al., 2011; Zeng et al., 2015). However, its efficacy and mechanisms in heat-stressed beef cattle require systematic elucidation.

This knowledge gap is particularly relevant for the Pinan cattle (Piedmontese × Nanyang), a breed renowned in central China for its rapid growth and high meat quality (Wei et al., 2022; Bo et al., 2024). Despite its economic importance, the physiological responses of this newly developed breed to heat stress and the efficacy of mitigants like OEO remain unexplored.

Therefore, this study provides the first comprehensive evaluation of OEO supplementation in Pinan cattle. We employed an integrated multi-omics approach, moving beyond conventional growth and antioxidant assessments to unravel the complex host-microbiota-metabolite interactions. Our goal was to determine the dose-dependent effects of OEO on heat-stress-impaired pathways and provide evidence-based recommendations for enhancing resilience in this unique breed.

## 2 Materials and methods

### 2.1 Experimental design and animal management

The experiment was conducted from June to August 2024. Thirty-six healthy 12-month-old Pinan (Piedmontese × Nanyang) bulls with uniform initial body weight (380 ± 25 kg) were obtained from the Xinye County Hengyong Beef Cattle Breeding Cooperative, Henan Province (32°17′N, 110°58′E). In a completely randomized design, the cattle were assigned to three dietary treatments (*n* = 12 cattle/treatment). The bulls were housed in a well-ventilated barn equipped with individual stalls, allowing for strict individual management, feeding, and supplementation. This design ensured that each animal received its precise dietary treatment without cross-contamination and that all measurements could be unequivocally attributed to the individual animal. Treatments were: (Nakamura and Morrison, 2010) CON, basal diet without OEO; (Mader et al., 2006) LOEO, basal diet + 7 g OEO/animal/day; (Morrison et al., 2014) HOEO, basal diet + 14 g OEO/animal/day. Following a 7-day dietary adaptation, all bulls entered a 60-day experimental period during which OEO supplementation was initiated and maintained as per treatment allocation.

Cattle were offered a total mixed ration (TMR) twice daily at 06:00 and 18:00 h. The daily OEO dose for each bull was first evenly mixed with a small quantity of the TMR (carrier), and then this premix was thoroughly incorporated into the individual’s entire 18:00 TMR portion using a mechanical mixer to ensure homogeneous distribution. This fixed feeding regimen (10 kg per feeding, 20 kg daily) was implemented to standardize nutrient and OEO intake. Feed refusals were recorded daily and were negligible (<5%), confirming that the actual OEO intake matched the intended dosage.

The oregano essential oil (OEO) used in this study was procured from Feidi Technology (Product Standard Code: Q/HFD0103-2023). Chemical characterization by gas chromatography–mass spectrometry (GC-MS) established that the oil was overwhelmingly dominated by phenolic compounds. The two primary bioactive phenols, thymol, and carvacrol, were identified as the major constituents, constituting 24.97% and 64.96% of the total composition, respectively. Their combined relative abundance of 89.93% definitively classifies this OEO as the carvacrol-thymol chemotype, which is renowned for its potent biological activities. Other notable components included D-limonene (6.49%) and 2,4-di-tert-butylphenol (1.79%), while the remaining identified compounds each accounted for less than 0.5% of the total profile.

The feed composition and nutritional components are shown in Table 1.

**Table 1:** Feed ingredient composition and nutritional content.

Ingredient	Composition (%)	Nutrient composition	Content
Corn	49.26	Dry matter (DM, %)	88.00
Soybean meal	19.70	Net energy for gain (NEg, MJ/kg)	5.80
DDGS	14.78	Crude protein (CP, %)	20.10
Palm kernel meal	7.88	Neutral detergent fiber (NDF, %)	35.50
Premix^a^	4.93	Acid detergent fiber (ADF, %)	21.60
Sodium bicarbonate	2.96	Calcium (Ca, %)	1.00
Salt	0.49	Phosphorus (P, %)	0.60
Total	100		

a The premix provided the following per kg of diet: Vitamin A 8,000 IU, Vitamin D₃ 2,500 IU, Vitamin E 50 IU; Fe 80 mg, Cu 10 mg, Mn 40 mg, Zn 60 mg, I 0.5 mg, Se 0.3 mg.

Given that the animals were individually stall-housed, fed a fixed amount, and supplemented precisely on an individual basis, the experimental unit for all subsequent statistical analyses was unequivocally defined as the individual animal.

### 2.2 Field Indicator measurement

#### 2.2.1 Environmental parameter measurements

At the daily heat-peak (14:00 h), ambient temperature (T, °C) and relative humidity (RH, %) were recorded at 1.5 m above the barn floor using a handheld digital thermo-hygrometer (TASI TA620A; accuracy ±0.5 °C and ±3% RH). The temperature–humidity index (THI) was calculated according to ASABE (citation number):


Thi=0.8T+RH(T−14.4)+46.4

Heat-stress severity was classified as THI ≤ 74 (no stress), 75–78 (mild), 79–83 (moderate), and ≥ 84 (severe) (Gaughan et al., 2008).

#### 2.2.2 Growth performance

On days 1 and 60, body weight was recorded at 06:00 following a 12-h fast (water allowed). Average daily gain (ADG) and feed conversion ratio (FCR) were calculated as:


$$\mathrm{ADG}(\mathrm{kg}\;d^{-1})=({\mathrm{BW}}_{\text{final}}-{\mathrm{BW}}_{\text{initial}})/60$$

FCR=daily feed intake/ADG

### 2.3 Sample collection and processing

Blood samples (5 mL) were collected from the tail vein into heparinized vacuum tubes. After 30 min clotting at 25 °C, samples were centrifuged (3,000 × *g*, 15 min, 131 4 °C). Serum was aliquoted (1 mL) into sterile cryovials, snap-frozen on dry ice (−78 °C), and stored at −40 °C pending untargeted metabolomics and biochemical analyses.

On the final day before morning feeding, ~100 mL rumen fluid was collected via an orogastric tube from each animal. Fluid was immediately filtered through four layers of sterile cheesecloth, and pH was recorded with a calibrated portable pH meter. Aliquots (~2 mL) of filtrate were snap-frozen in liquid nitrogen and stored at −80 °C pending 16S rRNA sequencing of the rumen microbiota.

Fresh fecal samples were obtained by digital rectal stimulation using sterile long-arm. In review gloves, snap-frozen in liquid nitrogen, and stored at −80 °C pending 16S rRNA sequencing of the gut microbiota.

### 2.4 Sample analysis

#### 2.4.1 Serum biochemical parameter assays

Serum concentrations of immunoglobulin A (IgA), immunoglobulin M (IgM), immunoglobulin G (IgG), total antioxidant capacity (T-AOC), total superoxide dismutase (T-SOD), glutathione peroxidase (GSH-Px), malondialdehyde (MDA), catalase (CAT), heat shock protein 70 (HSP70), cortisol (COR), triiodothyronine (T3), and thyroxine (T4) were determined using corresponding commercial assay kits (Nanjing Jiancheng Bioengineering Institute, China) in accordance with the manufacturer’s instructions.

The concentrations of alanine aminotransferase (ALT), aspartate aminotransferase (AST), alkaline phosphatase (ALP), urea (UREA), albumin (ALB), total protein (TP), glucose (GLU), triglycerides (TG), and free hemoglobin (Hb) were measured using a fully automated biochemical analyzer (Mindray BS-330E, Shenzhen Mindray Bio-Medical Electronics Co., Ltd., China).

#### 2.4.2 Serum untargeted metabolomics analysis

Serum untargeted metabolomics was performed by Personalbio Biotechnology Co., Ltd. (Shanghai, China). Ultra-high performance liquid chromatography coupled with high-resolution mass spectrometry (UHPLC-HRMS) analysis was performed using a Thermo Vanquish Flex UHPLC system (Thermo Fisher Scientific, USA) coupled to a Thermo Orbitrap Exploris 120 mass spectrometer (Thermo Fisher Scientific, USA).

Serum metabolites were extracted using a standardized protocol. Briefly, a 50 μL aliquot of each serum sample was mixed with 200 μL of pre-cooled methanol:acetonitrile (1:1, v/v). The mixture was vortexed for 30 s and then incubated at −20 °C for 30 min to precipitate proteins. After centrifugation at 12,000 × *g* and 4 °C for 10 min, 200 μL of the supernatant was collected and completely dried under a vacuum concentrator. The dried metabolite extract was reconstituted in 150 μL of 50% methanol containing 5 ppm L-2-chlorophenylalanine as an internal standard. The solution was vortexed for 30 s, centrifuged again under the same conditions, and the final supernatant was filtered through a 0.22 μm membrane prior to UHPLC-MS analysis.

A pooled quality control (QC) sample was generated by combining equal volumes of the filtrate from all individual samples. This QC sample was processed identically to the analytical samples and was injected repeatedly throughout the analytical sequence to monitor instrument stability and data quality.

Chromatographic separation was performed on an ACQUITY UPLC HSS T3 column (100Å, 1.8 μm, 2.1 mm × 100 mm; Waters, USA) maintained at 40 °C. The mobile phase flow rate was 0.4 mL/min, and the injection volume was 2 μL. The metabolites were detected using a Thermo Orbitrap Exploris 120 mass spectrometer (Thermo Fisher Scientific, USA) controlled by Xcalibur software (v4.7). The mass spectrometer was equipped with a HESI source and operated in both positive and negative ionization modes with a spray voltage of 3.5 kV and −3.0 kV, respectively. The sheath gas and auxiliary gas were set to 40 arb and 10 arb, respectively. The capillary temperature was 320 °C, and the auxiliary gas heater temperature was 300 °C. Full MS scans were acquired from 70 to 1,000 m/z at a resolution of 60,000. Data-dependent acquisition (DDA) mode was used for MS/MS fragmentation of the top 4 most intense ions at a resolution of 15,000.

Subsequent statistical analyses—including univariate analysis, multivariate analysis, and correlation analysis of differential metabolites—were conducted using the GenesCloud platform.^1^

#### 2.4.3 Rumen and gut microbiota analysis

Rumen fluid and fecal samples were shipped on dry ice to Personalbio Biotechnology In review Co., Ltd. (Shanghai, China) for 16S rRNA gene sequencing. After thawing and homogenization, total genomic DNA was extracted with the OMEGA Soil DNA Kit (M5635-02, Omega Bio-Tek, USA) following the manufacturer’s instructions. Extracted DNA was checked for integrity and fragment size on a 0.8% agarose gel, and concentration and purity were determined with a NanoDrop 2000/2000c spectrophotometer (Thermo Fisher Scientific, USA). High-quality DNA was used as template for PCR amplification of the V3–V4 hypervariable region of the bacterial 16S rRNA gene with primers 338F (5′-ACTCCTACGGGAGGCAGCA-3′) and 806R (5′-GGACTACHVGGGTWTCTAAT-3′). Amplicons were purified with AMPure XP beads and used to construct sequencing libraries. Libraries were sequenced on an Illumina NovaSeq 6000 platform (Illumina, USA) generating 2 × 150 bp paired-end reads. Raw sequencing reads were processed and analyzed as described below.

#### 2.4.4 Correlation analysis

Pearson correlation analysis was conducted to explore the relationships between differentially abundant gut/rumen microbes and differential serum metabolites. For each comparison, both the correlation coefficient (*r*) and the corresponding *p*-value are reported. To account for the multiple comparisons inherent in this high-dimensional data, the False Discovery Rate was controlled using the Benjamini–Hochberg procedure, and the resulting FDR-adjusted *p*-values (*q*-values) are reported. A correlation was considered statistically significant if the *q*-value was <0.05.

Furthermore, to aid in the biological interpretation of the magnitude of the correlations, the absolute value of the correlation coefficient *r* was used to define the strength of the relationship as follows: *r* ≥ 0.60: Strong correlation, 0.30 ≤ *r* < 0.60: Moderate correlation, *r* < 0.30: Weak correlation. Only correlations that were both statistically significant (*q* < 0.05) and at least of moderate strength (*r* ≥ 0.30) are discussed in the main text.

### 2.5 Data analysis and statistics

All data obtained from individual animals were analyzed using one-way analysis of variance (ANOVA) in SPSS software (Version 26.0). The individual animal was considered the experimental unit (*n* = 12 per treatment). The statistical model included the fixed effect of the dietary treatment (CON, LOEO, HOEO). When the ANOVA indicated a significant overall treatment effect (*p* < 0.05), differences between individual treatment means were further evaluated using the Least Significant Difference (LSD) *post hoc* test. Data are expressed as means ± standard deviation (SD) of the individual measurements. A probability value of *p* < 0.05 was considered statistically significant.

The individual animal was unequivocally considered the experimental unit for all statistical analyses (*n* = 12 per treatment), as treatments were applied, and all data (feed intake, blood parameters, microbial communities, etc.) were collected and measured on an individual basis.

## 3 Results

### 3.1 Effects of essential oil supplementation on growth performance of heat-stressed Pinan cattle

The Temperature–Humidity Index (THI), respiratory rate, and growth performance are summarized in Tables 2, 3. Throughout the trial, the mean THI exceeded 84 in all groups, indicating persistent severe heat stress. Respiratory rate did not differ among groups (*p* > 0.05). Likewise, initial body weight, final body weight, and average daily gain (ADG) were not significantly different (*p* > 0.05).

**Table 2:** Temperature, relative humidity, THI, and respiratory rate of each group of cattle house.

Items	Value (Mean ± SD)
Environmental parameters^a^	
Temperature (°C)	32.9 ± 2.9
Relative humidity	73.3 ± 9.2
THI	86.2 ± 3.0
Respiratory rate (breaths/min)^b^	
CON	33.9 ± 4.3
L-OEO	32.4 ± 6.2
H-OEO	30.6 ± 5.5
*p*-value	>0.05

a Environmental parameters were measured daily at 14:00 h and represent the overall conditions in the barn throughout the experimental period.

b Values are presented as mean ± SD by treatment group. The *p*-value from one-way ANOVA indicates no significant difference among groups.

**Table 3:** Effect of OEO on the ADG of Pinan cattle under heat stress.

Item	L-OEO	H-OEO	CON
Initial weight (kg)	249.50 ± 35.97	224.17 ± 47.28	211.67 ± 43.84
Final weight (kg)	266.67 ± 35.27	244.33 ± 43.91	242.17 ± 36.96
ADG (kg/d)	0.29 ± 0.25	0.34 ± 0.17	0.51 ± 0.38

### 3.2 Effects of OEO on serum biochemical parameters in heat-stressed Pinan cattle

As summarized in Table 4, 60 days of OEO supplementation significantly improved markers of liver injury, protein metabolism, and synthetic function. Compared with CON, both OEO-treated groups exhibited significantly lower serum alanine aminotransferase (ALT, *p* < 0.05) and aspartate aminotransferase (AST, *p* < 0.01) concentrations. Albumin (ALB) concentration was significantly higher in the low-dose group than in both the high-dose group (*p* < 0.01) and the control (*p* < 0.01). The high-dose group also exhibited significantly higher ALB than the control (*p* < 0.05). Total protein (TP) was also markedly elevated in L-OEO and H-OEO (*p* < 0.01). Furthermore, urea (UREA) concentration was significantly reduced in L-OEO (*p* < 0.05) and H-OEO (*p* < 0.01) relative to CON.

**Table 4:** Effect of OEO on the serum biochemical indicators of Pinan cattle under heat stress.

Item	L-OEO	H-OEO	CON
ALP (U/mL)	35.32 ± 3.08	37.67 ± 0.71	38.42 ± 1.29
ALT (U/mL)	52.08 ± 3.56^b^	57.82 ± 3.75^b^	75.07 ± 5.89^a^
AST (U/mL)	130.93 ± 2.74^B^	134.14 ± 2.36^B^	156.02 ± 5.96^A^
ALB (g/L)	33.22 ± 0.59^Aa^	30.43 ± 0.39^b^	28.53 ± 0.88^B^
TP (g/L)	49.63 ± 0.91^A^	48.42 ± 0.59^A^	41.40 ± 2.01^B^
UREA (U/mL)	1.89 ± 0.09^b^	1.72 ± 0.12^B^	2.38 ± 0.10^Aa^
GLU (U/mL)	3.28 ± 0.14^ab^	2.95 ± 0.16^b^	3.89 ± 0.41^a^
TG (U/mL)	0.17 ± 0.02^b^	0.24 ± 0.03^b^	0.31 ± 0.03^a^
IgA (U/mL)	290.48 ± 6.81^A^	278.70 ± 4.06^A^	253.56 ± 7.64^B^
IgM (U/mL)	1311.77 ± 22.85^a^	1295.21 ± 25.63^a^	1215.55 ± 24.46^b^
IgG (U/mL)	1493.08 ± 73.78	1519.50 ± 57.86	1350.39 ± 65.84
HSP-70 (U/mL)	195.70 ± 3.64	197.42 ± 5.82	179.22 ± 12.31
GSH-Px (U/mL)	59.00 ± 2.32^B^	127.55 ± 16.67^A^	33.27 ± 6.75^B^
SOD (U/mL)	19.55 ± 0.39^a^	19.43 ± 0.35^a^	17.83 ± 0.44^b^
T-AOC (U/mL)	1.14 ± 0.17	1.84 ± 0.28	1.35 ± 0.24
CAT (U/mL)	8.41 ± 1.67^a^	5.37 ± 1.04^ab^	3.43 ± 0.76^b^
MDA (nmol/mL)	3.87 ± 0.79^Cc^	8.53 ± 0.96^b^	13.33 ± 2.01^Aa^
Hb (mg/L)	83.99 ± 11.03^ab^	54.97 ± 7.86^b^	147.51 ± 34.16^a^
Cor (ng/mL)	131.25 ± 25.48	138.99 ± 15.67	154.66 ± 11.32
T3 (pmol/L)	10.28 ± 1.39	11.08 ± 1.76	9.05 ± 1.70
T4 (nmol/L)	188.97 ± 13.04^ab^	196.60 ± 12.88^a^	174.19 ± 11.06^b^
T3/T4	0.05 ± 0.01	0.06 ± 0.01	0.05 ± 0.01

Within the same row, values with different uppercase superscript letters indicate highly significant differences (*p* < 0.01), while those with different lowercase superscript letters indicate significant differences (*p* < 0.05). The absence of superscript letters or the same letters denotes no significant difference (*p* > 0.05). The same convention applies to the following tables.

Regarding immune function, OEO supplementation significantly enhanced early immune responses. Serum IgA concentrations were significantly higher in both L-OEO and H-OEO compared to CON (*p* < 0.01), and IgM concentrations were significantly higher than those in CON (*p* < 0.05).

In terms of antioxidant capacity, H-OEO showed a significant increase in GSH-Px activity relative to both L-OEO and CON (*p* < 0.01). SOD activity was significantly higher in both OEO-supplemented groups than in the control (*p* < 0.05). CAT activity in L-OEO was significantly higher than in CON (*p* < 0.05), whereas no significant difference was observed between H-OEO and either L-OEO or CON (*p* > 0.05).

L-OEO showed the lowest MDA, differing from both CON (*p* < 0.01) and H-OEO (*p* < 0.05), while H-OEO was also lower than CON (*p* < 0.05). For other serum biochemical indicators, the hemoglobin (Hb) concentration in H-OEO was significantly lower than that in CON (*p* < 0.05). In contrast, the thyroxine (T4) concentration in H-OEO was significantly higher than in CON (*p* < 0.05). For both Hb and T4, no significant difference was detected between H-OEO and L-OEO (*p* > 0.05).

### 3.3 Serum untargeted metabolomics

The untargeted metabolomics analysis detected a total of 31,747 metabolite features in the serum samples, with 18,816 features identified in positive ion mode and 12,931 features in negative ion mode. While a significant proportion of these features could not be unequivocally assigned to known metabolic pathways in current databases, they represent a comprehensive snapshot of the systemic metabolic state. The identified metabolites were dominated by features consistent with lipid and phospholipid species (e.g., various phosphatidylcholines detected primarily in positive ion mode), amino acid derivatives, and other organic acids. This extensive coverage of the serum metabolome provides a robust basis for detecting relative changes in metabolite abundance between experimental groups.

#### 3.3.1 Principal component analysis (PCA)

**Figure 1:**
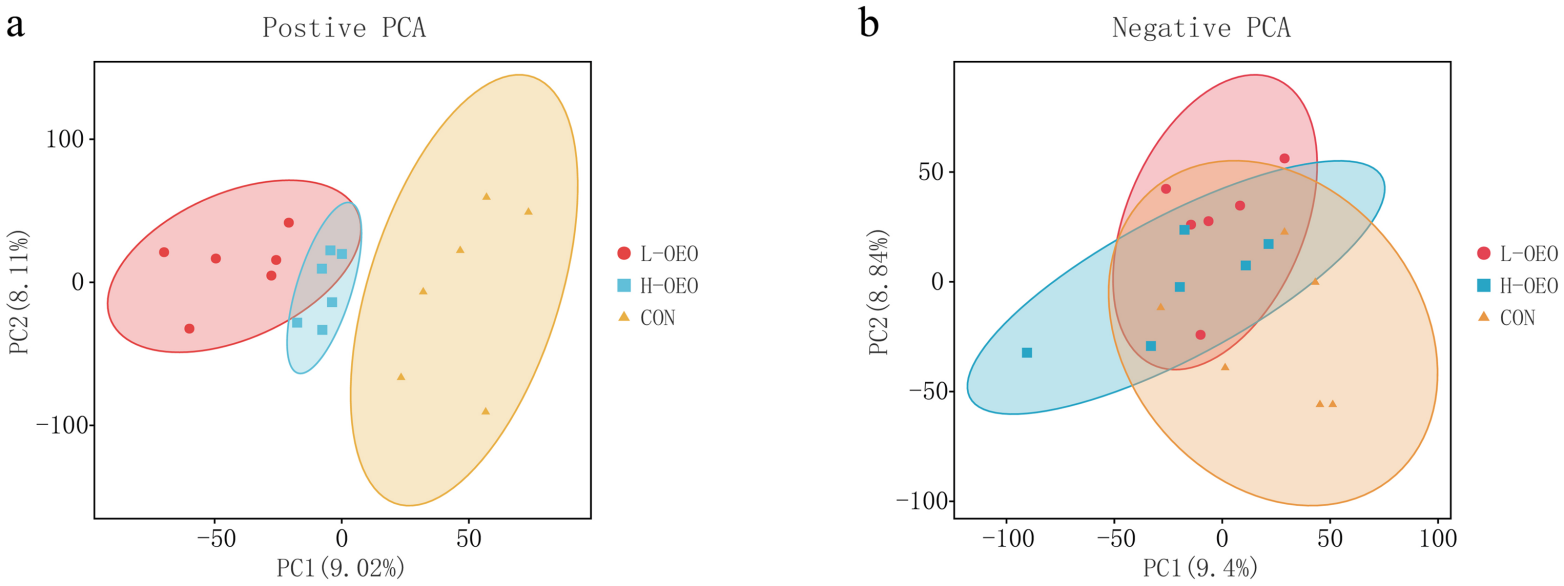
PCA analysis of serum metabolites.

As shown in Figure 1, under positive ion mode, a clear separation in metabolic profiles was observed between both OEO-supplemented groups (L-OEO and H-OEO) and the control group (CON), whereas the metabolic profiles of L-OEO and H-OEO were not clearly separated from each other. In contrast, no obvious separation was detected among any of the groups under negative ion mode.

#### 3.3.2 Differential metabolite screening

**Figure 2:**
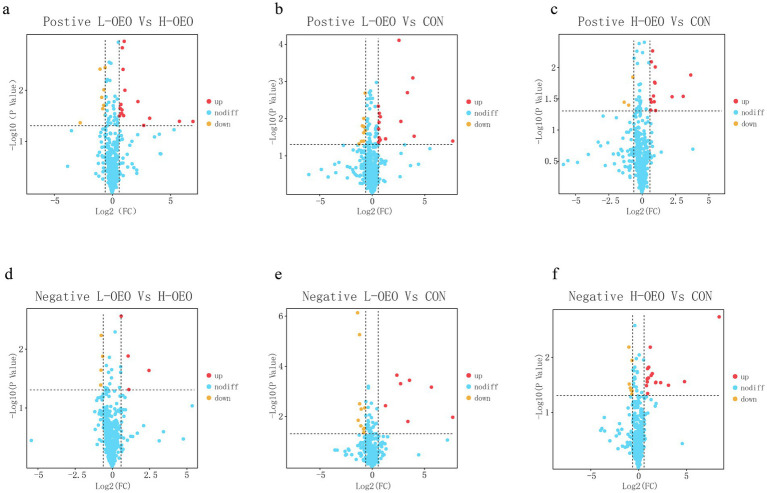
Volcano plot for screening differential metabolites in the serum of heat stressed Pinang cattle with different doses of OEO. **(a)** Positive ion mode: L-OEO vs. H-OEO. **(b)** Positive ion mode: L-OEO vs. CON. **(c)** Positive ion mode: H-OEO vs. CON. **(d)** Negative ion mode: L-OEO vs. H-OEO. **(e)** Negative ion mode: L-OEO vs. CON. **(f)** Negative ion mode: H-OEO vs. CON.

Differential metabolites were identified based on the criteria of *p* < 0.05 and fold change (FC) > 1.5 or FC < 0.58, and were visualized using volcano plots (Figure 2). The results indicated that the differential metabolites between L-OEO and H-OEO consisted primarily of various phosphatidylcholines (PCs). Comparisons between L-OEO and CON revealed significant alterations in metabolites including multiple PCs, isobutyrylglycine, N-acetylvaline, hydroxyphenylacetylglycine, 2-methylbutyrylglycine (2-MBG), thymol sulfate, and docosahexaenoic acid ethyl ester. Meanwhile, differential metabolites between H-OEO and CON included isobutyrylglycine, N-acetylvaline, hydroxyphenylacetylglycine, thymol sulfate, glycodeoxycholic acid, taurochenodeoxycholic acid, and cholylalanine.

To gain a systemic overview of the metabolic perturbations, KEGG pathway enrichment analysis was performed on the differential metabolites identified in all pairwise comparisons (L-OEO vs. CON, H-OEO vs. CON, and L-OEO vs. H-OEO). Notably, no pathways met the threshold for statistical significance (FDR-corrected *p*-value < 0.05) in any comparison. This indicates that the metabolic response to OEO supplementation was not characterized by the strong enrichment or depletion of any single canonical pathway. Instead, the effects appear to be distributed across a broad network of interconnected metabolic processes, as reflected in the diverse classes of metabolites altered (e.g., acylglycines, bile acids, and phospholipids).

### 3.4 16S microbiomics

#### 3.4.1 Microbial composition

The effects of OEO supplementation on the ruminal and gut microbiota are summarized in Tables 5–8. OEO administration exerted distinct modulatory effects on the microbial communities in the rumen and intestine.

**Table 5:** Effects of OEO on the abundance of phylum-level gut microbiota in heat-stressed Pinan cattle.

Item	L-OEO	H-OEO	CON
*Firmicutes_A*	0.544 ± 0.024	0.58 ± 0.035	0.567 ± 0.039
*Bacteroidota*	0.345 ± 0.025	0.323 ± 0.03	0.333 ± 0.032
*Firmicutes_D*	0.038 ± 0.005	0.038 ± 0.009	0.039 ± 0.012
*Spirochaetota*	0.036 ± 0.025	0.022 ± 0.009	0.016 ± 0.009
*Patescibacteria*	0.013 ± 0.006	0.008 ± 0.005	0.008 ± 0.005
*Firmicutes_C*	0.005 ± 0.003	0.005 ± 0.003	0.007 ± 0.005
*Actinobacteriota*	0.005 ± 0.002	0.005 ± 0.002	0.007 ± 0.003
*Verrucomicrobiota*	0.002 ± 0.001^b^	0.004 ± 0.001^b^	0.007 ± 0.003^a^
*Proteobacteria*	0.003 ± 0.001	0.002 ± 0.001	0.003 ± 0.001
*Cyanobacteria*	0.001 ± 0.001	0.002 ± 0.001	0.002 ± 0.001
Others	0.009 ± 0.004	0.012 ± 0.006	0.01 ± 0.003

**Table 6:** Effects of OEO on the abundance of phylum-level rumen microbiota in heat-stressed Pinan cattle.

Item	L-OEO	H-OEO	CON
*Bacteroidota*	0.656 ± 0.03^a^	0.568 ± 0.052^b^	0.579 ± 0.039^b^
*Firmicutes_A*	0.152 ± 0.032^B^	0.224 ± 0.028^A^	0.229 ± 0.025^A^
*Firmicutes_C*	0.081 ± 0.024	0.082 ± 0.038	0.068 ± 0.01
*Firmicutes_D*	0.048 ± 0.009	0.052 ± 0.013	0.057 ± 0.028
*Patescibacteria*	0.019 ± 0.005	0.027 ± 0.013	0.022 ± 0.005
*Spirochaetota*	0.008 ± 0.002	0.008 ± 0.003	0.01 ± 0.003
*Verrucomicrobiota*	0.008 ± 0.002	0.008 ± 0.003	0.007 ± 0.002
*Actinobacteriota*	0.007 ± 0.006	0.007 ± 0.002	0.007 ± 0.002
*Fibrobacterota*	0.007 ± 0.002^a^	0.005 ± 0.002^b^	0.005 ± 0.001^b^
*Proteobacteria*	0.004 ± 0.002	0.002 ± 0.001	0.003 ± 0.002
Others	0.011 ± 0.003	0.016 ± 0.005	0.013 ± 0.002

**Table 7:** Effects of OEO on the abundance of genus-level gut microbiota in heat-stressed Pinan cattle.

Item	L-OEO	H-OEO	CON
*Faecousia*	0.136 ± 0.023	0.134 ± 0.016	0.137 ± 0.03
*Cryptobacteroides*	0.128 ± 0.016^a^	0.108 ± 0.011^b^	0.098 ± 0.012^b^
*Paraprevotella*	0.035 ± 0.007	0.039 ± 0.007	0.042 ± 0.016
*Alistipes_A*	0.036 ± 0.008	0.033 ± 0.008	0.035 ± 0.008
*Phocaeicola_A*	0.026 ± 0.008	0.025 ± 0.008	0.029 ± 0.004
*SFMI01*	0.021 ± 0.003	0.025 ± 0.005	0.022 ± 0.003
*UBA737*	0.025 ± 0.006	0.021 ± 0.004	0.019 ± 0.005
*Onthenecus*	0.02 ± 0.005	0.019 ± 0.004	0.017 ± 0.005
*CAG-41*	0.016 ± 0.005	0.019 ± 0.005	0.019 ± 0.006
*Treponema_D*	0.028 ± 0.024	0.012 ± 0.005	0.01 ± 0.008
Others	0.529 ± 0.032	0.565 ± 0.028	0.571 ± 0.028

**Table 8:** Effects of OEO on the abundance of genus-level rumen microbiota in heat-stressed Pinan cattle.

Item	L-OEO	H-OEO	CON
*Prevotella*	0.313 ± 0.35^A^	0.161 ± 0.04^B^	0.182 ± 0.045^B^
*Cryptobacteroides*	0.071 ± 0.007^B^	0.104 ± 0.011^A^	0.11 ± 0.019^A^
*Limimorpha*	0.067 ± 0.023	0.101 ± 0.039	0.083 ± 0.013
*Succiniclasticum*	0.068 ± 0.021	0.068 ± 0.031	0.057 ± 0.012
*UBA1711*	0.045 ± 0.016	0.041 ± 0.01	0.038 ± 0.007
*RF16*	0.044 ± 0.017	0.036 ± 0.019	0.039 ± 0.015
*Sodaliphilus*	0.023 ± 0.011^b^	0.028 ± 0.004^ab^	0.038 ± 0.015^a^
*Paraprevotella*	0.02 ± 0.004	0.026 ± 0.008	0.025 ± 0.006
*Nanosyncoccus*	0.012 ± 0.004	0.014 ± 0.006	0.013 ± 0.005
*UBA4334*	0.022 ± 0.005^A^	0.008 ± 0.004^B^	0.008 ± 0.003^B^
Others	0.314 ± 0.039	0.412 ± 0.033	0.407 ± 0.042

At the phylum level, the dominant bacterial phyla in the gut were *Firmicutes_A* and *Bacteroidota*, which collectively accounted for over 90% of the total bacterial abundance. The relative abundance of *Verrucomicrobiota* was significantly lower in both OEO-supplemented groups (L-OEO and H-OEO) than in the CON (*p* < 0.05). No significant differences were observed among the groups for the remaining phyla (*p* > 0.05).

In the rumen, the predominant bacterial phyla were similarly *Bacteroidota* and *Firmicutes_A*. Compared to CON, L-OEO exhibited a significant increase in the abundance of *Bacteroidota* (*p* < 0.05) and a highly significant reduction in *Firmicutes_A* (*p* < 0.01). In contrast, no significant differences in microbial composition were observed between H-OEO and the CON (*p* > 0.05). Moreover, the abundance of *Fibrobacterota* was significantly higher in L-OEO than in both H-OEO and CON.

At the genus level, *Faecalicoccus* and *Cryptobacteroides* were the dominant genera in the gut across all groups. The abundance of *Cryptobacteroides* was significantly higher in L-OEO than in H-OEO and C (*p* < 0.05).

In the rumen, the predominant genera were *Prevotella* and *Cryptobacteroides*. Compared with H-OEO and CON, L-OEO showed a significantly higher abundance of *Prevotella* (*p* < 0.01). Conversely, the abundance of *Cryptobacteroides* was markedly lower in L-OEO than in both H-OEO and CON (*p* < 0.01). Additionally, the abundance of *Sodaliphilus* was significantly lower in L-OEO than in CON (*p* < 0.05), while no significant difference was observed between L-OEO and H-OEO (*p* > 0.05).

#### 3.4.2 Microbial diversity

**Figure 3:**
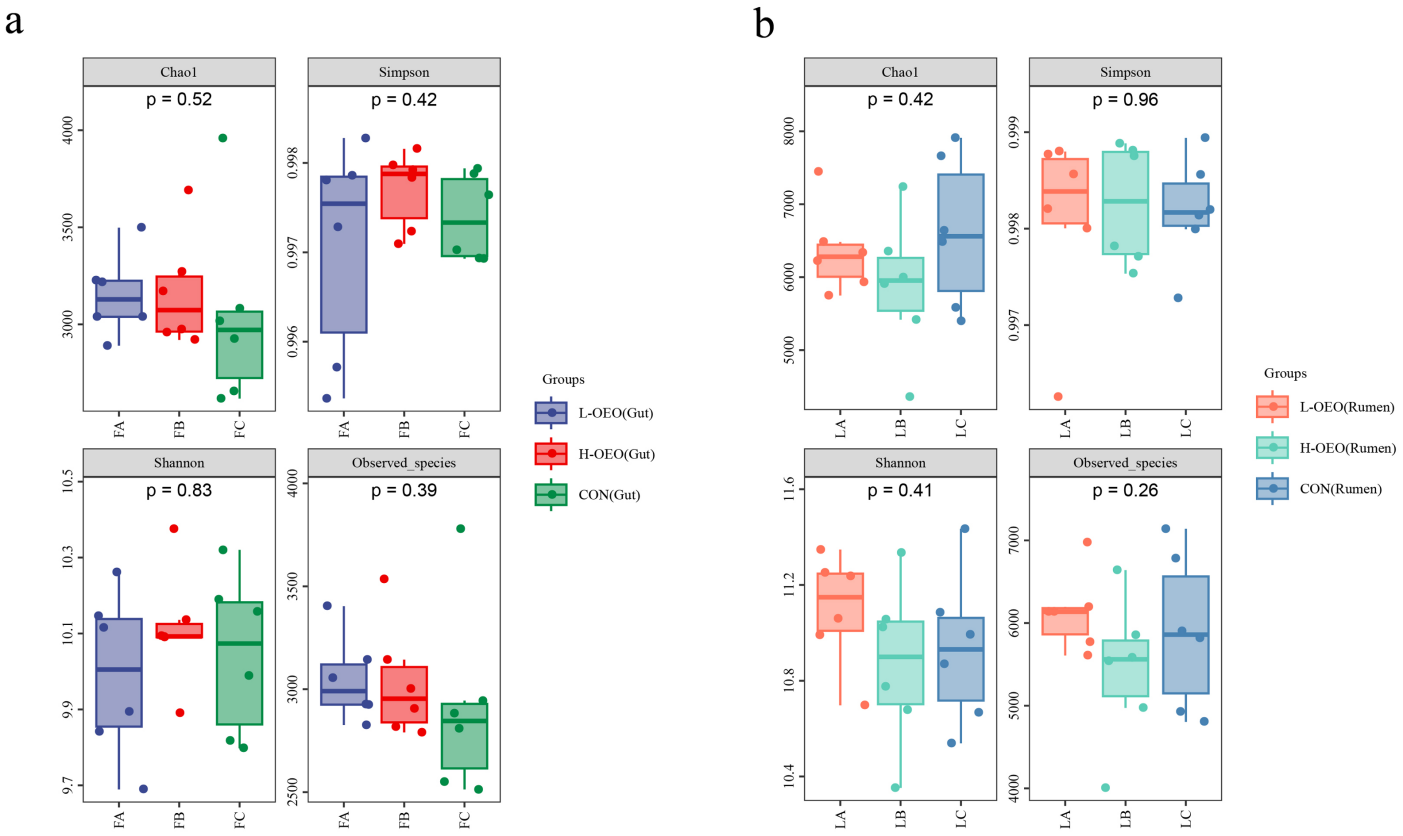
Alpha diversity analysis of gut and ruminal microbiota. **(a)** Alpha diversity analysis of gut microbiota. **(b)** Alpha diversity analysis of ruminal microbiota.

Analysis of *α*-diversity indices (Chao1 and Shannon) revealed no significant differences among the three treatment groups in either the ruminal or gut microbiota (all *p* > 0.05; Figure 3), indicating that OEO supplementation did not alter the overall richness or evenness of the microbial communities.

**Figure 4:**
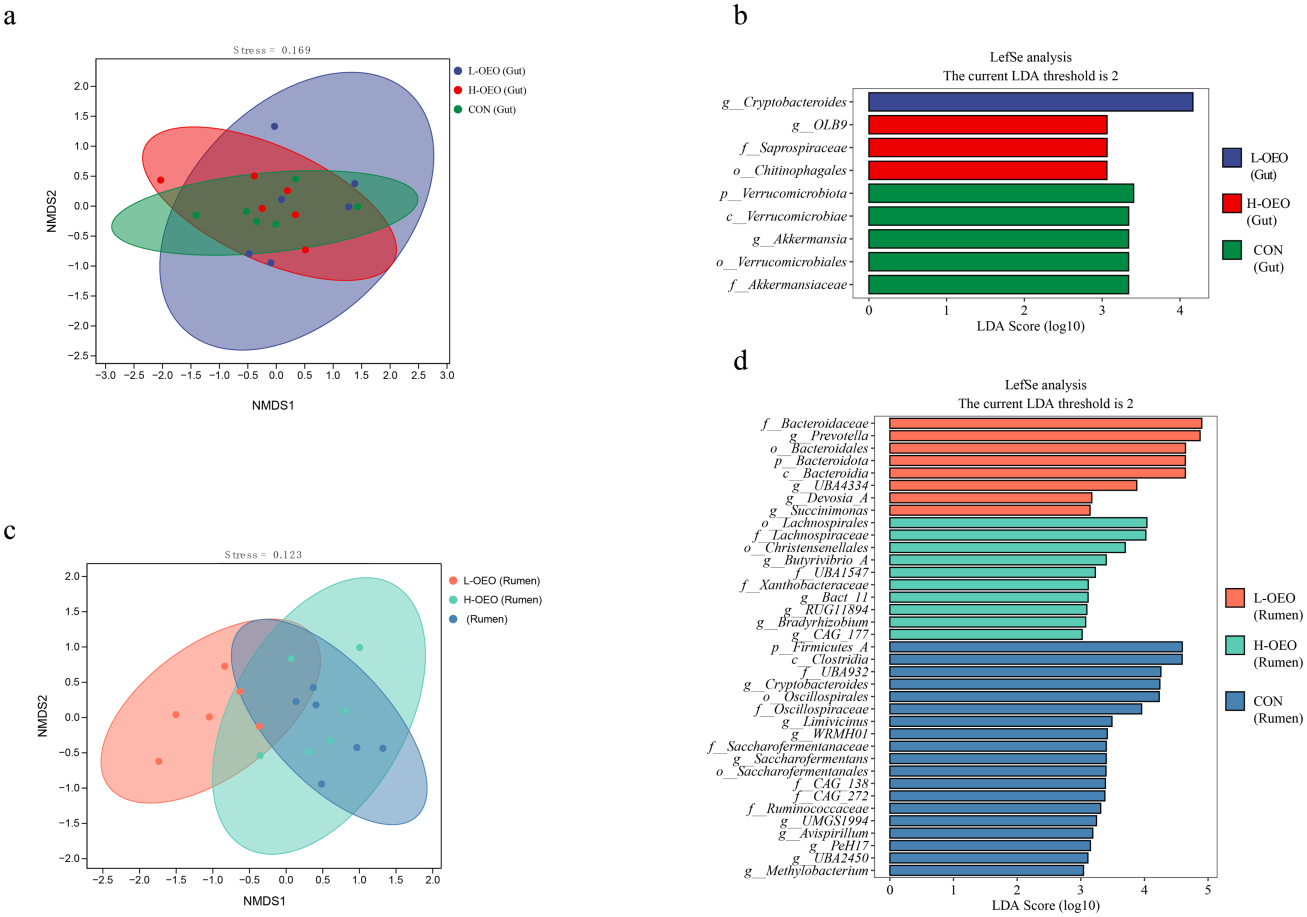
NMDS analysis and LDA score plots of gut and rumen microbiota. **(a)** NMDS of gut microbiota. **(b)** LEfSe results for differential gut microbial taxa. Bar length represents log_10_-transformed LDA score. **(c)** NMDS of rumen microbiota. **(d)** LEfSe results for differential rumen bacterial taxa. Bar length represents log_10_-transformed LDA score.

Beta diversity of the gut and rumen microbiota was assessed using non-metric multidimensional scaling (NMDS). As illustrated in Figure 4, no distinct clustering pattern was observed among the groups in the gut microbial communities. In contrast, clear separation was evident in the rumen microbiota, with samples from L-OEO and H-OEO forming clusters distinct from those of CON.

Linear discriminant analysis (LDA) revealed that in the gut, the genus *Cryptobacteroides* was significantly enriched in the lL-OEO. No characteristic microbial taxa were identified as significantly enriched in the H-OEO, while the phylum *Verrucomicrobiota* was significantly enriched in the cCON.

In the rumen, low-dose OEO supplementation resulted in significant enrichment of *Bacteroidaceae* and *Prevotella*. In contrast, high-dose OEO supplementation was associated with significant enrichment of *Lachnospirales* and *Butyrivibrio*.

To infer whether the observed compositional shifts in the microbiota translated to changes in their metabolic potential, we performed phylogenetic investigation of communities by reconstruction of unobserved states (PICRUSt2) analysis. The predicted abundances of Kyoto Encyclopedia of Genes and Genomes (KEGG) pathways were compared across the three dietary groups. No KEGG pathways at Level 3 were found to be significantly different between any of the group comparisons (CON vs. L-OEO, CON vs. H-OEO, or L-OEO vs. H-OEO) after false discovery rate (FDR) correction for multiple comparisons (all *q* > 0.05). This indicates that while OEO supplementation altered the relative abundance of specific bacterial taxa, it did not induce a major restructuring of the core predicted functional potential of the microbial community at the resolution of this analysis.

### 3.5 Integrated analysis

**Figure 5:**
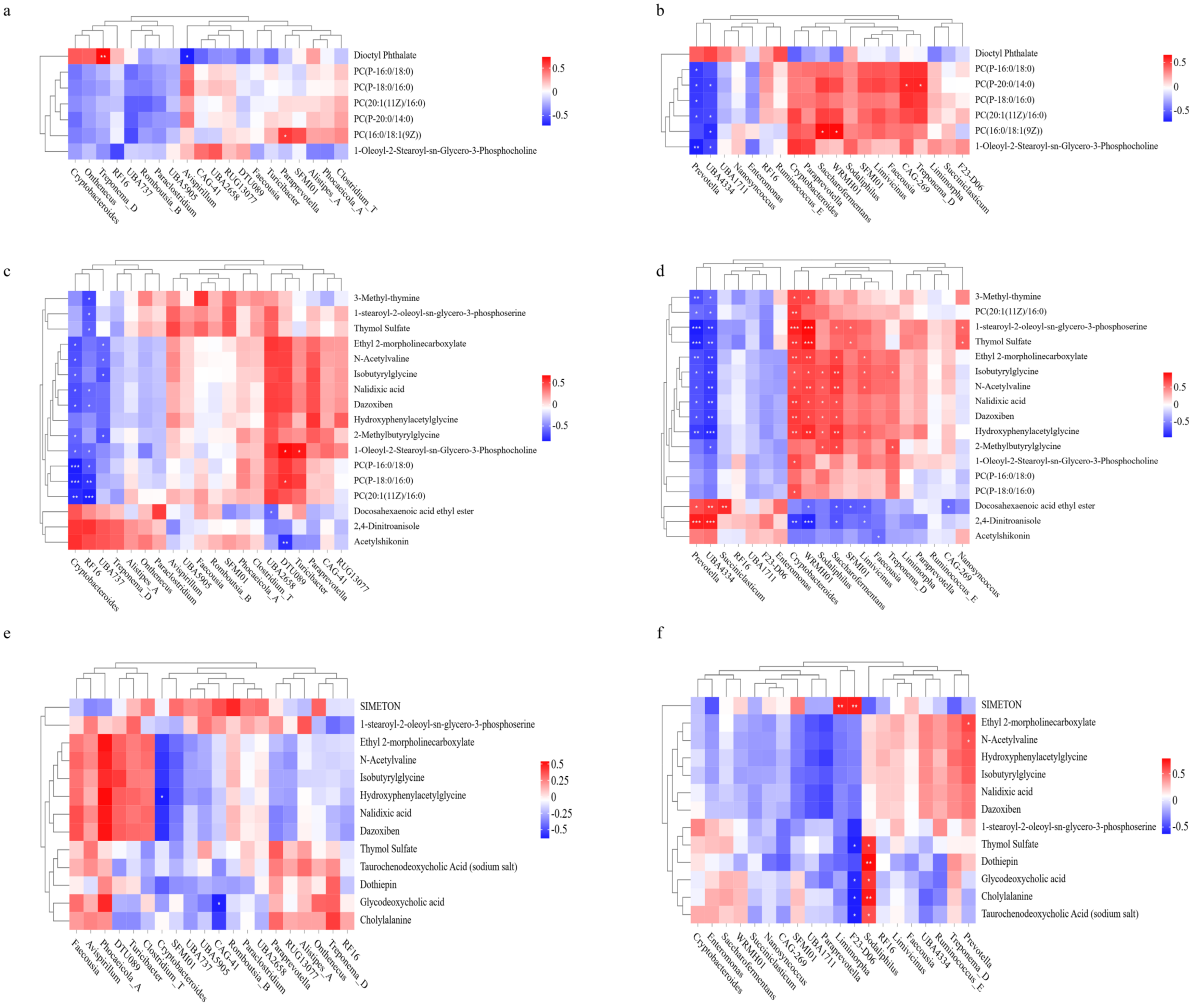
Correlation analysis of differential gut and rumen microbes with differential serum metabolites. **(a)** Correlation analysis of differential gut microbes and serum metabolites between L-OEO and H-OEO. **(b)** Correlation analysis of differential rumen microbes and serum metabolites between L-OEO and L-OEO. **(c)** Correlation analysis of differential gut microbes and serum metabolites between L-OEO and CON. **(d)** Correlation analysis of differential rumen microbes and serum metabolites between L-OEO and CON. **(e)** Correlation analysis of differential gut microbes and serum metabolites between H-OEO and CON. **(f)** Correlation analysis of differential rumen microbes and serum metabolites between H-OEO and CON.

Pearson correlation analysis was conducted to explore the relationships between differentially abundant gut/rumen microbes and serum metabolites across the experimental groups. As illustrated in Figure 5, within the gut microbiota, serum concentrations of phosphatidylcholine (PC), hydroxyphenylacetylglycine, and isobutyrylglycine showed significant negative correlations (*p* < 0.05) with the abundance of *Cryptobacteroides* (gut) when comparing L-OEO and CON In the rumen, a significant negative correlation (*p* < 0.05) was identified between serum PC levels and the abundance of *Prevotella* (rumen) in both L-OEO and H-OEO. Furthermore, in the comparison between L-OEO and CON, serum levels of PC, N-acetylvaline, hydroxyphenylacetylglycine, and isobutyrylglycine were significantly negatively correlated (*p* < 0.05) with *Prevotella* (rumen) abundance, but significantly positively correlated (*p* < 0.05) with the abundance of *Cryptobacteroides* (rumen) and *Saccharofermentans* (rumen). Conversely, serum docosahexaenoic acid ethyl ester concentration was positively correlated (*p* < 0.05) with *Prevotella* (rumen) and negatively correlated (*p* < 0.05) with *Saccharofermentans* (rumen). Between H-OEO and CON, serum thymol sulfate exhibited a significant positive correlation (*p* < 0.05) with the abundance of *Sodaliphilus* (rumen), while serum N-acetylvaline was significantly negatively correlated (*p* < 0.05) with *Prevotella* (rumen).

To mitigate the risk of false positives from multiple comparisons, we performed FDR correction (Benjamini–Hochberg method) on all Pearson correlation analyses between microbes and metabolites. After rigorous correction, robust microbe-metabolite associations were identified in both the intestine and rumen. The most prominent association network emerged from comparing the rumen between the L-OEO and CON groups, where key bacterial genera (e.g., Prevotella, Cryptobacteroides, UBA4334) showed extremely strong correlations with multiple phospholipid and amino acid metabolites (all *r* > 0.75, *q* < 0.01). All significant associations are detailed in Table 9.

**Table 9:** Significant microbiota-metabolite correlations after FDR correction.

Comparison	Habitat	Microorganism	Metabolite	*r*-value	*q*-value
L-OEO vs. H-OEO	Gut	*Paraprevotella*	PC [16:0/18:1(9Z)]	0.617	0.032
L-OEO vs. CON	Gut	*Cryptobacteroides*	PC (P-16:0/18:0)	−0.837	0.001
L-OEO vs. CON	Gut	*Cryptobacteroides*	PC (P-18:0/16:0)	−0.825	0.002
L-OEO vs. CON	Gut	*Cryptobacteroides*	PC [20:1(11Z)/16:0]	−0.784	0.005
L-OEO vs. CON	Gut	*UBA737*	Isobutyrylglycine	−0.666	0.036
L-OEO vs. CON	Gut	*RF16*	3-Methyl-thymine	−0.706	0.021
L-OEO vs. CON	Gut	*RF16*	PC [20:1(11Z)/16:0]	−0.833	0.002
H-OEO vs. CON	Gut	*CAG-41*	Glycodeoxycholic acid	−0.614	0.02
L-OEO vs. H-OEO	Rumen	*Prevotella*	1-Oleoyl-2-Stearoyl-sn-Glycero-3-Phosphocholine	−0.708	0.032
L-OEO vs. CON	Rumen	*Prevotella*	1-stearoyl-2-oleoyl-sn-glycero-3-phosphoserine	−0.922	<0.001
L-OEO vs. CON	Rumen	*Prevotella*	Thymol Sulfate	−0.889	<0.001
L-OEO vs. CON	Rumen	*Cryptobacteroides*	PC [20:1(11Z)/16:0]	0.756	0.001
L-OEO vs. CON	Rumen	*Cryptobacteroides*	1-stearoyl-2-oleoyl-sn-glycero-3-phosphoserine	0.846	0.001
L-OEO vs. CON	Rumen	*Succiniclasticum*	Docosahexaenoic acid ethyl ester	0.811	0.003
L-OEO vs. CON	Rumen	*UBA4334*	Hydroxyphenylacetylglycine	−0.867	0.001
L-OEO vs. CON	Rumen	*UBA4334*	1-stearoyl-2-oleoyl-sn-glycero-3-phosphoserine	−0.813	0.002
L-OEO vs. CON	Rumen	*UBA4334*	Isobutyrylglycine	−0.788	0.005
L-OEO vs. CON	Rumen	*UBA4334*	N-Acetylvaline	−0.803	0.003
L-OEO vs. CON	Rumen	*UBA4334*	2-Methylbutyrylglycine	−0.864	0.028
L-OEO vs. CON	Rumen	*UBA4334*	Docosahexaenoic acid ethyl ester	0.751	0.001
L-OEO vs. CON	Rumen	*WRMH01*	3-Methyl-thymine	0.686	0.028
L-OEO vs. CON	Rumen	*WRMH01*	1-stearoyl-2-oleoyl-sn-glycero-3-phosphoserine	0.931	<0.001
L-OEO vs. CON	Rumen	*WRMH01*	Hydroxyphenylacetylglycine	0.808	<0.001
L-OEO vs. CON	Rumen	*WRMH01*	Thymol Sulfate	0.893	<0.001
H-OEO vs. CON	Rumen	*Sodaliphilus*	Cholylalanine	0.754	0.018

## 4 Discussion

Heat stress reduces feed intake, increases respiratory rate, and disrupts ruminal In review fermentation in Pinan cattle, collectively leading to a negative energy balance. Concurrently, high ambient temperatures promote lipid peroxidation of the inner mitochondrial membrane and enhance electron leakage from Complexes I and III, thereby increasing superoxide anion (O₂^−^•) production. In addition, suppressed ATP synthase activity lowers energy production efficiency. These metabolic alterations collectively trigger excessive reactive oxygen species (ROS) generation (Khan et al., 2021), resulting in widespread oxidative damage.

Under heat stress, reduced feed intake is normally the primary constraint on weight gain. By fixing daily intake at 20 kg TMR per head we removed this constraint; consequently, the numerical rise in ADG (L-OEO 0.29 H-OEO 0.34 CON 0.51 kg/d) reflects a true improvement in the efficiency with which dietary nutrients were converted to live-weight. It should be noted that the L-OEO began the trial significantly heavier than both the H-OEO and CON. Because initial body weight is a key determinant of subsequent growth potential, heavier animals typically exhibit a flatter growth curve and devote a larger fraction of energy to maintenance. Thus, the fact that L-OEO still achieved a higher ADG than CON, despite its higher starting weight and associated maintenance burden, underscores the positive biological impact of the low-dose essential-oil supplement.

Analysis of serum biochemical parameters indicated that OEO supplementation significantly reduced markers of liver injury (ALT and AST) and protein catabolism (UREA), while enhancing indicators of synthetic function (ALB and TP). These results suggest that OEO alleviates heat stress-induced hepatic damage by protecting hepatocytes and improving protein metabolism (Cui et al., 2024; Song et al., 2014; Zhou et al., 2020). Notably, the L-OEO showed greater efficacy in promoting protein synthesis, whereas the H-OEO resulted in a more pronounced reduction in blood urea nitrogen.

Consistent with the results of the present study, in which OEO improved liver function in heat-stressed cattle, Zhang et al. (2024) reported that dietary OEO supplementation in sows also exerted significant hepatoprotective effects by enhancing serum antioxidant capacity (significantly increasing CAT and GSH), reducing the level of the liver-specific enzyme AST, and optimizing the structure of the gut microbiota.

In terms of immune response, OEO supplementation significantly elevated early immune mediators (IgM) and mucosal immunity-related antibodies (IgA), but did not significantly affect longer-term immunoglobulins (IgG). These observations are consistent with the established immune shift under heat stress, which suppresses Th1-mediated cellular immunity while promoting Th2-driven humoral responses. This suggests that OEO may counter heat-induced immunosuppression primarily through enhancement of the humoral immune pathway (Bernabucci et al., 2010; Gupta et al., 2022).

Consistent with the findings of Luo et al. (2024), dietary supplementation with oregano essential oil under summer feeding conditions significantly increased serum immunoglobulin (IgA and IgM) levels in calves by modulating the ruminal microbiota, suggesting that oregano essential oil can effectively enhance humoral immunity in ruminants under heat stress.

Regarding antioxidant capacity, the L-OEO exhibited a significant reduction in the lipid peroxidation product MDA and an increase in CAT activity, whereas the H-OEO demonstrated a pronounced enhancement in GSH-Px activity. This dose-dependent variation may reflect the concentration-related bioactivity of carvacrol and thymol, the primary active components of OEO (Nehme et al., 2021). The simultaneous increase in SOD activity further supports the synergistic mitigation of oxidative stress, although overall antioxidant capacity did not improve significantly. In review the absence of a significant change in COR levels suggests that OEO may help attenuate overactivation of the hypothalamic–pituitary–adrenal (HPA) axis.

The significant increase in T4 observed in the H-OEO suggests the involvement of thyroid hormone in the metabolic regulation mediated by OEO supplementation. Meanwhile, the decrease in free Hb indicates enhanced erythrocyte membrane stability (Das et al., 2016), which is consistent with the overall improvement in antioxidant status.

These findings indicate that OEO effectively mitigates liver injury, improves metabolic function, enhances immune responses, and bolsters antioxidant capacity in heat-stressed Pinan cattle. The benefits are likely mediated by thymol and carvacrol, the principal bioactive of OEO, which donate electrons to neutralize superoxide anions, providing direct antioxidant protection and upregulating key enzymes (GSH-Px, SOD, CAT). Consequently, the endogenous antioxidant defense system is comprehensively reinforced. The divergent responses between low- and high-dose supplementation suggest that lower dosage may be optimal for specific physiological endpoints.

Notably, the improvements in antioxidant and immune parameters occurred in the absence of significant changes in respiratory rate, a conventional clinical indicator of heat stress. This apparent discrepancy can be explained by the distinct levels of biological organization at which these measurements are taken and the primary mechanisms of action of OEO. Respiratory rate is a whole-animal, integrative physiological response coordinated by the hypothalamic thermoregulatory center to maximize evaporative heat loss (West, 2003). Its modulation is often a refractory endpoint that may not respond until core body temperature is significantly altered. In contrast, the antioxidant and immune parameters measured (e.g., SOD, CAT, IgA) reflect the cellular and biochemical milieu, which are more directly influenced by dietary bioactive compounds like thymol and carvacrol.

The observed divergence suggests that the beneficial effects of OEO supplementation in heat-stressed cattle may operate primarily at the sub-physiological level. By directly scavenging ROS and potentially upregulating the expression of endogenous antioxidant enzymes, OEO mitigates oxidative damage at its source within tissues. Similarly, its immunomodulatory effects are likely mediated through direct interactions with immune cells and the gut-associated lymphoid tissue. This intracellular and molecular improvement can occur independently of, and potentially prior to, changes in the animal’s effort to dissipate heat. Therefore, the enhancement of the antioxidant and immune systems represents a more sensitive and early indicator of OEO’s efficacy, underscoring its role in fortifying the host’s internal defense mechanisms against the cellular assaults of heat stress, even when the external stressor (high THI) persists and the overall physiological heat-dissipation response remains fully engaged.

Previous studies have shown that heat stress increases insulin, which in turn suppresses lipolysis and curtails lipid mobilization (Abbas et al., 2020; Ma et al., 2021; Delgado-Martin et al., 2024). To compensate for the ensuing energy deficit, muscle protein is catabolized to release branched-chain amino acids (BCAAs) such as valine and isoleucine. These BCAAs are subsequently metabolized in the liver by branched-chain amino acid transaminase (BCAT) and branched-chain *α*-keto acid dehydrogenase (BCKDH). This process drives deamination pathways—leucine → isovaleryl-CoA → 3-methylcrotonyl-CoA → acetyl-CoA, and valine → succinyl-CoA—thereby furnishing substrates for the tricarboxylic acid (TCA) cycle and sustaining energy production.

In the present study, BCAA-derived acylglycines—N-acetylvaline, isobutyrylglycine, and 2-methylbutyrylglycine (2-MBG)—together with hydroxyphenylacetylglycine, 3-methyl-thymine (3-MT), and thymol sulfate, were all significantly elevated. The accumulation of acylglycines points to impaired hepatocellular detoxification, most likely owing to oxidative inhibition of glycine N-acyltransferase (GLYAT); this inhibition mirrors both liver dysfunction and accelerated muscle proteolysis (Guo et al., 2020; Alves et al., 2019). Elevated 2-MBG further signals disrupted isoleucine catabolism (Newgard et al., 2009), which may curtail TCA-cycle flux and ATP synthesis. Detection of thymol sulfate, a phase-II conjugate of thymol, provides an indirect indicator of the parent compound’s in-vivo antioxidant and anti-inflammatory activity (Zhou et al., 2020; Yu et al., 2016; Placha et al., 2022).

These alterations underscore a metabolic vicious cycle under heat stress: BCKDH inhibition blocks BCAA oxidation to succinyl-CoA, curtailing TCA-cycle entry and causing intermediate accumulation (Sun et al., 2016). Simultaneously, oxidative stress depletes cysteine, the rate-limiting precursor of glutathione (GSH) (Ulrich and Jakob, 2019; Nguyen et al., 2023), perpetuating the In review cycle: energy deficit drives BCAA catabolism; BCAA depletion impairs GSH synthesis; weakened antioxidant defense exacerbates mitochondrial damage and ROS generation; and further compromise of energy metabolism ensues (Sharma et al., 2024; Circu and Aw, 2010; Kim et al., 2015; Bifari and Nisoli, 2017).

In the H-OEO, bile acid-related compounds—glycodeoxycholic acid, taurochenodeoxycholic acid and cholylalanine—were also markedly up-regulated alongside the BCAA-derived metabolites. Glycodeoxycholic acid, a secondary bile acid, controls lipid metabolism and bile acid homeostasis via the FXR–FGF19 pathway (Meessen et al., 2025), but can exert pro-inflammatory effects in settings such as acute pancreatitis (Fauzi et al., 2022). Taurochenodeoxycholic acid displays anti-inflammatory and immunomodulatory activity through up-regulation of SRSF9 and potentiation of glucocorticoid receptor (GR) signaling (Bao et al., 2021). Cholylalanine, a conjugated bile acid, may modulate nuclear receptor activity and reshape the gut microbiota (Zhao et al., 2025).

The KEGG enrichment analysis, while not identifying any single significantly altered pathway, provides an important systemic perspective. The absence of a starkly dysregulated pathway suggests that OEO does not act like a targeted pharmaceutical agent that blocks a specific enzymatic reaction. Instead, its supplementation in heat-stressed cattle facilitates a global rebalancing of metabolism that cuts across multiple pathway boundaries.

This finding is entirely consistent with the known pleiotropic nature of plant essential oils. Rather than overwhelming a single pathway, the components of OEO, particularly thymol and carvacrol, likely exert subtle modulatory effects on multiple cellular targets—including membrane integrity, enzyme activity, and signal transduction—which collectively manifest as the observed improvements in liver health, antioxidant status, and immune function (Walasek-Janusz et al., 2024; Cui et al., 2024). The most sensitive biomarkers of this system-wide rebalancing were the specific metabolite changes we detected, such as the accumulation of acylglycines signaling a relief of hepatic detoxification pressure and the presence of thymol sulfate confirming the direct bioactivity of OEO constituents. Thus, the mechanism of action is best described as a “systems-level stabilization” rather than a “pathway-specific intervention”.

Collectively, our data show that oregano essential oil (OEO) markedly reshapes metabolism in heat-stressed Pinan cattle. By accelerating branched-chain amino acid (BCAA) catabolism, the supplement partially offsets the energy deficit; however, concomitant inhibition of branched-chain *α*-keto acid dehydrogenase (BCKDH) causes intermediate metabolites to accumulate and exacerbates oxidative injury. Moreover, high-dose OEO activates bile-acid metabolism, potentially providing an alternative energy source during thermal stress (Van Passel et al., 2011).

The rumen and gut microbial communities in ruminants form a complex and highly integrated ecosystem that plays a vital role in nutrient metabolism, immune defense, developmental regulation, and environmental adaptation. In the present study, at the phylum level, only *Verrucomicrobiota* showed a significant decrease in abundance in the gut of treated animals. *Akkermansia muciniphila*, a representative species of this phylum, utilizes mucin as its sole carbon and nitrogen source by secreting glycoside hydrolases, sulfatases, and carbohydrate esterases to degrade mucin (Van Passel et al., 2011). The decline in its abundance is likely attributable to oregano-essential-oil-induced disintegration of the cell envelope, as thymol and carvacrol insert into lipid bilayers and destabilise membrane integrity, consequently reducing *Verrucomicrobiota* populations.

In the rumen, supplementation enriched *Bacteroidota* and *Fibrobacterota* while depressing *Firmicutes_A*. *Bacteroidota* drive polysaccharide degradation (cellulose, starch) (Hao et al., 2021), and their expansion likely improves dietary energy harvest. *Fibrobacterota*, the primary cellulolytic rumen taxon (Yan et al., 2024), directly boosts forage conversion. *Firmicutes_A harbours* numerous glycoside hydrolases and sugar transporters (e.g., *Lachnospiraceae*) that cleave complex polysaccharides and import monosaccharides; consequently, its decline may lower intestinal carbohydrate uptake efficiency (Mohamed, 2025).

At the genus level, only low-dose OEO increased gut *Cryptobacteroides*. Members of this genus encode a broad suite of carbohydrate-active enzymes (CAZymes) that target complex hemicelluloses (Lin et al., 2024), implying improved energy harvest from the diet. In review in the rumen, the same dose elevated *Prevotella* while depressing *Sodaliphilus* and *Cryptobacteroides*. Although *Prevotella* hydrolyses cellulose and protein, its overgrowth can raise ruminal ammonia nitrogen (Chiquette et al., 2008). These dose-dependent shifts underscore the fine-tuning of microbial function and host metabolic homeostasis by OEO.

Both *Cryptobacteroides* and *Sodaliphilus* extensive polysaccharide-utilization loci (PULs) that cleave complex polysaccharides into simple sugars, boosting fiber digestion (Lin et al., 2024). Their decline likely reflects direct membrane disruption by OEO bioactive—thymol and carvacrol—which compromise cell-wall integrity.

A key finding of our microbiome analysis is the dissociation between the significant compositional shifts (*β*-diversity and specific taxa) and the stability observed in both *α*-diversity and predicted metagenomic functions (PICRUSt2). The absence of *α*-diversity changes demonstrates that OEO, at the doses tested, acts as a precision modulator of the gut ecosystem rather than a broad-spectrum disruptor. It selectively enriches beneficial taxa (e.g., *Prevotella*) without compromising the overall ecological stability.

The specific enrichment of *Bacteroidaceae* and *Prevotella* under low-dose OEO supplementation can be attributed to a combination of direct and indirect mechanisms. Firstly, certain members of the *Bacteroidetes* phylum, particularly *Prevotella*, possess efflux pump systems and membrane adaptations that may confer a degree of intrinsic tolerance to low concentrations of plant secondary compounds, allowing them to thrive when more sensitive taxa are mildly inhibited (Song et al., 2014; Zhang et al., 2021).

Secondly, and perhaps more importantly, the primary bioactive components of OEO, thymol and carvacrol, are known to disrupt the membrane of Gram-negative bacteria. At sub-inhibitory (low) concentrations, this membrane perturbation may not be lethal but can instead act as an environmental cue or stressor. This can trigger a shift in microbial metabolism towards energy storage and competitive growth. *Prevotella* and other *Bacteroidetes* are adept at rapidly uptaking and fermenting soluble sugars and starches. The mild stress from low-dose OEO might channel microbial metabolism towards the efficient degradation of readily available polysaccharides, a niche where these bacteria excel.

Indirectly, by creating a mildly selective environment, low-dose OEO likely reduces competition from OEO-sensitive bacteria (e.g., some *Firmicutes*), freeing up ecological space and resources. This allows resilient and metabolically versatile taxa like *Prevotella* to dominate. This enriched community is highly proficient at degrading dietary polysaccharides, leading to increased propionate production, which aligns with our observed improvements in host energy metabolism.

In contrast, the lack of additional benefits and the onset of potential adverse effects at the high OEO dose (14 g/d) can be mechanistically explained by a shift from a selective, stimulatory effect to a broad-spectrum antimicrobial one. The ‘hormetic effect’—where a low dose is beneficial while a high dose is detrimental—is well-documented for plant essential oils (Zhou et al., 2019; Hristov et al., 2013).

At high concentrations, the phenolic compounds thymol and carvacrol exert their full antimicrobial power by: 1. Integrating into and severely disrupting microbial membranes, causing irreversible damage, leakage of cellular contents, and cell death. This non-selective action does not spare beneficial, functionally important bacteria. 2. Quenching microbial quorum-sensing signals, thereby disrupting the microbial cross-talk necessary for a stable and cooperative ecosystem.

This broad-spectrum suppression is evidenced in our study by the absence of characteristic beneficial taxa enrichment in the H-OEO and the minimal impact on ruminal community structure. The collapse of the delicate symbiotic network, particularly the suppression of key fibrolytic bacteria, could impair the digestion of structural carbohydrates (e.g., cellulose and hemicellulose), potentially explaining the numerical (though not significant) reduction in energy-producing bacteria.

Furthermore, the high dose may have direct negative effects on the host. The rumen epithelium itself can be irritated by high concentrations of potent phenols, potentially compromising its absorptive and barrier functions. Additionally, an excessive dose may overwhelm the host’s liver detoxification pathways, increasing the metabolic burden rather than alleviating it. This shift from a precision-guided modulation at low dose to an indiscriminate suppression at high dose provides a clear mechanistic rationale for the observed “threshold effect,” where doubling the dosage failed to yield additional benefits and instead showed a tendency to suppress some positive physiological responses.

Microbial-diversity analysis revealed high resilience of the gut microbiota to OEO, whereas the rumen community responded in a dose-dependent manner: low-dose OEO significantly improved ruminal structure, but high-dose had minimal impact. Linear discriminant analysis (LDA) identified gut *Cryptobacteroides* as significantly enriched under low-dose OEO, likely enhancing energy harvest through degradation of complex polysaccharides (Sidney et al., 2024). No characteristic taxa were detected in the H-OEO, consistent with a broad-spectrum inhibitory effect or microbial adaptive resistance at elevated OEO concentrations (Zhou et al., 2020). In controls, *Verrucomicrobiota* was markedly enriched; members of this phylum encode GH29 (fucosidase), GH141 (rhamnosidase) and sulfotransferases that specialize in degrading sulfated polysaccharides such as fucoidan (Orellana et al., 2022).

Low-dose OEO supplementation significantly enriched *Bacteroidaceae* and *Prevotella* in the rumen, suggesting enhanced polysaccharide degradation and propionate production. This metabolic shift may compete for H₂, thereby reducing the substrate availability for methanogens and potentially mitigating methane emissions. In contrast, high-dose OEO enriched *Lachnospirales* and *Butyrivibrio*, which are core taxa involved in hemicellulose-pectin degradation and butyrate production via PUL-enzyme systems and the butyrate kinase pathway. These metabolic activities not only provide energy to the host but also contribute to milk fat synthesis (Wirth et al., 2018; Palevich et al., 2019). The ruminal microbiota in the CON was predominantly composed of Firmicutes and *Ruminococcaceae*, which specialize in degrading lignin and cellulose. This process produces H₂ and butyrate, thereby supplying substrates to methanogens (Malik et al., 2023).

The lack of significant PICRUSt2 results, despite clear taxonomic changes, suggests that the primary mechanism behind the improved host physiology may not be a major rewiring of microbial *catabolic potential*. Instead, the benefits are likely mediated through alternative pathways: firstly, via direct host effects of the absorbed bioactive OEO compounds (thymol and carvacrol), which our serum biochemistry and metabolomics strongly support; and secondly, through potentially fine-tuned changes in microbial gene expression or metabolic output that are not captured by a static genetic potential prediction. This holistic view underscores that the health benefits of phytogenic feed additives can be achieved without destabilizing the functional core of the gut microbiota.

In summary, OEO induced only minor gut-microbiota shifts: low-dose enrichment of *Cryptobacteroides* improved energy harvest, whereas the high dose lacked discernible benefits, plausibly owing to broad-spectrum inhibition or microbial resistance. In the rumen, a clear dose–response prevailed: the low dose selectively expanded polysaccharide-degrading and propionate-producing taxa, boosting energy yield and curtailing methanogenesis; the high dose favored fiber degraders and elevated butyrate. These results underscore the need for precise dosing when using OEO to refine rumen fermentation and mitigate methane emissions in cattle.

In addition to altering the composition of intestinal and rumen microbial communities, our analysis of microbiota–metabolite correlations, following strict FDR correction, revealed the mechanisms by which these microbial changes influence host metabolic status.

In the intestinal context, a significant positive correlation was observed between *Paraprevotella* and PC [16:0/18:1(9Z)] in both the L-OEO and H-OEO. This suggests that *Paraprevotella* may participate in or promote this phospholipid metabolic pathway (Yao et al., 2022), indicating that L-OEO and H-OEO treatments may influence host phospholipid metabolism by modulating *Paraprevotella*, with a more pronounced effect at higher concentrations.

In the L-OEO and CON, *Cryptobacteroides* showed a significant negative correlation with several phospholipids (PCs). This implies that *Cryptobacteroides* may inhibit the synthesis or promote the degradation of these phospholipids, suggesting that L-OEO treatment may influence phospholipid metabolism by downregulating *Cryptobacteroides*. These phospholipids may be associated with cell membrane structure or signal transduction (O’Donnell, 2022).

Moreover, *UBA737* was significantly negatively correlated with Isobutyrylglycine, suggesting that *UBA737* may inhibit the metabolism of Isobutyrylglycine. *RF16* exhibited significant negative correlations with 3-Methyl-thymine and PC [20:1(11Z)/16:0], indicating that *RF16* may be involved in the negative regulation of pyrimidine metabolism and phospholipid metabolism. Thus, L-OEO treatment may affect DNA synthesis and cell membrane function by modulating *RF16* (Liu et al., 2019; Li et al., 2024).

In the H-OEO and CON, *CAG-41* was significantly negatively correlated with Glycodeoxycholic acid, suggesting that *CAG-41* may inhibit the metabolism of glycodeoxycholic acid. *CAG-41*belongs to *Firmicutes_A*, many members of which are known to possess bile salt hydrolase (BSH) activity and are capable of deconjugating bile acids, thereby altering bile acid composition and concentration (Ridlon et al., 2006). This indicates that H-OEO treatment may influence host bile acid metabolism by modulating *CAG-41*, subsequently affecting lipid absorption or intestinal barrier function.

In the rumen, within the L-OEO and H-OEO, *Prevotella* showed a significant negative correlation with 1-Oleoyl-2-Stearoyl-sn-Glycero-3-Phosphocholine. *Prevotella bivia* has been experimentally confirmed to secrete PLA₂ or other phospholipases capable of hydrolyzing PC into lysophosphatidylcholine and free fatty acids (Mikamo et al., 1998). It is inferred that *Prevotella* may inhibit the synthesis or promote the degradation of this phospholipid, suggesting that both L-OEO and H-OEO treatments might influence ruminal phospholipid metabolism by modulating *Prevotella*, with the higher dose exhibiting a more pronounced inhibitory effect.

In the L-OEO and CON, *Prevotella* was significantly negatively correlated with 1-stearoyl-2-oleoyl-sn-glycero-3-phosphoserine (*r* = −0.922, *q* = 0) and Thymol Sulfate (*r* = −0.889, *q* = 0). A *mucosal desulfating sulfatase (mdsA)* gene was cloned and expressed from *Prevotella* strain RS2 isolated from pig colon, and its product can remove sulfate from sugars or mucins (Wright et al., 2000). It is hypothesized that *Prevotella* may inhibit the metabolism of Thymol Sulfate, and L-OEO treatment might affect rumen membrane lipid structure and sulfate detoxification processes by downregulating *Prevotella*.

*Cryptobacteroides* was significantly positively correlated with PC [20:1(11Z)/16:0] (*r* = 0.756, *q* = 0.001) and 1-stearoyl-2-oleoyl-sn-glycero-3-phosphoserine (*r* = 0.846, *q* = 0.001). *Cryptobacteroides* may synthesize choline, a precursor for PC synthesis (Broad and Dawson, 1976; Sommese et al., 2012), suggesting it could promote the synthesis or accumulation of these phospholipids. This indicates that L-OEO treatment might influence ruminal phospholipid metabolism by upregulating *Cryptobacteroides* – a finding contrary to the intestinal results, highlighting site-specific effects.

*Succiniclasticum* was significantly positively correlated with Docosahexaenoic acid ethyl ester (DHA-EE) (*r* = 0.811, *q* = 0.003). *Succiniclasticum* may be involved in the synthesis or metabolism of DHA-EE, suggesting that L-OEO treatment could affect rumen unsaturated fatty acid metabolism (Carreño et al., 2019) by modulating *Succiniclasticum*, thereby influencing host lipid nutrition.

*UBA4334* showed significant negative correlations with Hydroxyphenylacetylglycine, 1-stearoyl-2-oleoyl-sn-glycero-3-phosphoserine, Isobutyrylglycine, N-Acetylvaline, and 2-Methylbutyrylglycine. *UBA4334* might inhibit the metabolism of these amino acids and phospholipids. L-OEO treatment could potentially impact ruminal amino acid metabolism and membrane lipid synthesis by downregulating *UBA4334*. Further isolation and genomics-centered characterization of the *UBA4334* strain are necessary to validate this causal relationship.

*WRMH01* was significantly positively correlated with 3-Methyl-thymine (*r* = 0.686, *q* = 0.028), 1-stearoyl-2-oleoyl-sn-glycero-3-phosphoserine (*r* = 0.931, *q* = 0), Hydroxyphenylacetylglycine (*r* = 0.808, *q* = 0), and Thymol Sulfate (*r* = 0.893, *q* = 0). *WRMH01* may promote pyrimidine, phospholipid, and sulfate metabolism. L-OEO treatment might influence ruminal DNA synthesis, membrane lipid structure, and detoxification processes by upregulating *WRMH01*. Further isolation and identification of the *WRMH01* strain are required to confirm the causal link.

In the H-OEO and CON, *Sodaliphilus* was significantly positively correlated with Cholylalanine (*r* = 0.754, *q* = 0.018). However, no publicly available studies have directly reported that *Sodaliphilus* can promote or participate in Cholylalanine metabolism, indicating that further research is still needed.

In summary, both L-OEO and H-OEO influence phospholipid, bile acid, amino acid, and fatty acid metabolism in both the intestine and rumen by modulating specific microorganisms (such as *Paraprevotella*, *Cryptobacteroides*, and *Prevotella*). H-OEO demonstrates superior efficacy in regulating phospholipid metabolism in the intestine and exhibits stronger inhibition of phospholipid degradation in the rumen. In contrast, L-OEO exerts opposite effects in the rumen compared to the intestine (e.g., in the regulation of *Cryptobacteroides*), highlighting site-specific effects. Further studies are needed to validate the causal relationships for certain microbiota–metabolite associations (e.g., those involving *UBA4334* and *WRMH01*).

While this study provides clear evidence for the dose-dependent effects of OEO in heat-stressed cattle, certain limitations should be considered. Firstly, although the individual stall-feeding design was ideal for precisely controlling OEO intake, the number of biological replicates (*n* = 12 per treatment), while standard for such studies, inherently limits the statistical power to detect more subtle effects. Future studies with a larger sample size would be valuable to confirm and extend our findings. Secondly, the fixed feeding regimen (20 kg/head/day), while crucial for standardizing nutrient and OEO intake, means that the potential interplay between OEO supplementation and voluntary feed intake under heat stress could not be assessed. Investigations involving ad libitum feeding are needed to explore this interaction and its implications for practical production settings.

## 5 Conclusion

This study provides the first systematic evaluation of the dose-dependent effects of oregano essential oil (OEO) on Pinan cattle under sustained severe heat stress. The results demonstrate that a low dose of 7 g/animal/day optimally enhances liver function, antioxidant capacity, and immune response, while enriching beneficial ruminal taxa such as Prevotella and Bacteroidota to support energy harvest. Notably, low-dose OEO also differentially regulates phospholipid and amino acid metabolism across intestinal and ruminal environments, reflecting site-specific microbial modulation. In contrast, doubling the dosage to 14 g/animal/day not only failed to yield additional benefits but also induced broad-spectrum microbial inhibition and potential disruption of host metabolic homeostasis, revealing a clear “threshold effect.” Therefore, 7 g/animal/day is recommended as a precise and effective supplementation level of OEO for alleviating heat stress in Pinan cattle. Future studies should validate its economic and environmental potential in production settings under ad libitum feeding and explore synergistic models combining OEO with fermentable energy sources.

## Data Availability

The datasets presented in this study can be found in online repositories. The names of the repository/repositories and accession number(s) can be found at: gaotengyun@henau.edu.cn.

## Footnotes

1
https://www.genescloud.cn
